# Development of Neutralization Assay Using an eGFP Chikungunya Virus

**DOI:** 10.3390/v8070181

**Published:** 2016-06-28

**Authors:** Cheng-Lin Deng, Si-Qing Liu, Dong-Gen Zhou, Lin-Lin Xu, Xiao-Dan Li, Pan-Tao Zhang, Peng-Hui Li, Han-Qing Ye, Hong-Ping Wei, Zhi-Ming Yuan, Cheng-Feng Qin, Bo Zhang

**Affiliations:** 1 Key Laboratory of Special Pathogens and Biosafety, Center for Emerging Infectious Diseases, Wuhan Institute of Virology, Chinese Academy of Sciences, Wuhan 430071, China; Dengchenglin29@126.com (C.-L.D.); luckyliu_110@163.com (S.-Q.L.); Xulinlin.2007yahoo@163.com (L.-L.X.); zhangpantao123@163.com (P.-T.Z.); lipenghui101@126.com (P.-H.L.); yehq@wh.iov.cn (H.-Q.Y.); hpwei@wh.iov.cn (H.-P.W.); yzm@wh.iov.cn (Z.-M.Y.); 2 Ningbo International Travel Healthcare Center, Ningbo 315012, China; zhoudg@nbciq.gov.cn; 3 School of Medicine, Hunan Normal University, Changsha 410000, China; lxd@live.cn; 4 State Key Laboratory of Pathogen and Biosecurity, Beijing Institute of Microbiology and Epidemiology, Beijing 100071, China; chengfeng_qin@126.com

**Keywords:** Chikungunya virus, reporter virus, antiviral, neutralization antibody

## Abstract

Chikungunya virus (CHIKV), a member of the *Alphavirus* genus, is an important human emerging/re-emerging pathogen. Currently, there are no effective antiviral drugs or vaccines against CHIKV infection. Herein, we construct an infectious clone of CHIKV and an eGFP reporter CHIKV (eGFP-CHIKV) with an isolated strain (assigned to Asian lineage) from CHIKV-infected patients. The eGFP-CHIKV reporter virus allows for direct visualization of viral replication through the levels of eGFP expression. Using a known CHIKV inhibitor, ribavirin, we confirmed that the eGFP-CHIKV reporter virus could be used to identify inhibitors against CHIKV. Importantly, we developed a novel and reliable eGFP-CHIKV reporter virus-based neutralization assay that could be used for rapid screening neutralizing antibodies against CHIKV.

## 1. Introduction

Chikungunya virus (CHIKV) is an important mosquito-borne virus that belongs to the genus *Alphavirus*, family *Togaviridae*. The complete viral genome consists of a linear, positive-sense, single-stranded RNA around 12 kilobases (Kb) [1,2]. The 5′ two-thirds of the genome encodes the nonstructural proteins (nsP1-nsP2-nsP3-nsP4) that were required for replication and transcription of the viral genome in the cytoplasm of infected cells. The structural proteins (C-E3-E2-6K-E1) that are responsible for viral particles formation are translated from a subgenomic (sg) messenger RNA. The sg RNA is transcribed from the remaining 3′ one-third of the genome with a 26S promoter that is present on the full-length negative-stranded RNA replication intermediate.

CHIKV mainly causes abrupt high fever, headache, rashes, myalgia, and arthralgia [3,4], and occasionally the arthritis may last for months or years. Although it is historically regarded as a none life-threatening pathogen [5], the large outbreak of CHIKV on the Island of La Réunion in 2005 indicated that the virus may have become more virulent for humans [6]. Furthermore, lethal cases of encephalitis have also been reported for CHIKV infection [6]. CHIKV is primarily transmitted by *Aedes aegypti* mosquitoes. Recently, it was shown that A226V mutation in the structural protein E1 significantly increased fitness of the virus for *Ae. albopictus* mosquitoes and likely contributed to the epidemic [7]. So far, CHIKV has resulted in numerous outbreaks and been considered as a re-emerging pathogen [1,2,8]. The importation of CHIKV to mainland China was firstly reported in 2008 [9], and the first documented outbreak of CHIKV in China occurred in 2010 in Guangdong province [10,11].

Currently, there are no effective antiviral treatments and vaccines against CHIKV infection. Although mechanisms of protective immunity against CHIKV are poorly understood, different vaccine strategies [12,13,14,15,16,17] and monoclonal antibodies (mAbs) [18,19] that neutralized CHIKV were developed. A reliable neutralization (NT) assay is an important method to validate the efficiency of potential vaccines during vaccine development, and is also essential for screening of neutralizing mAbs. Furthermore, NT assay is also necessary to determine the immune status of a patient.

Traditionally, plaque reduction neutralization tests (PRNTs) and inhibition of the cytopathogenic effects (CPEs) [20] were used for NT assay. Recently, some new methods based on the non-infectious virus replicon particles (VRPs) and the pseudotyped lentiviral vector have been developed for the CHIKV NT assay [21,22]. CHIKV VRPs were produced by co-transfecting BHK-21 cells with a CHIKV replicon expressing Gaussia luciferase (Gluc) and two helper RNAs expressing the CHIKV capsid and other structural proteins, respectively [21]. The CHIKV-pseudotyped lentiviral vector was prepared by co-transfection of plasmids encoding the CHIKV glycoproteins E3, E2, 6K, and E1, packaging elements, and a luciferase reporter [22]. The luciferase activities were used as readout to determine CHIKV neutralization sera/antibodies for both assays [21,22].

In this study, we developed another convenient alternative NT assay for CHIKV. We firstly constructed an infectious clone of CHIKV and a stable eGFP reporter CHIKV (eGFP-CHIKV) with a newly isolated strain of Asian lineage. The eGFP-CHIKV replicated efficiently that was comparable with wild type CHIKV (WT CHIKV) and genetically stable at least after five rounds of viral passages. Using the known inhibitor of CHIKV, we confirmed that the expression levels of eGFP could be used to quantify the replication of CHIKV. Based on eGFP-CHIKV reporter virus, a new neutralization assay for detection of CHIKV neutralizing serum/antibody was developed. Our method is a rapid and quantitative assay for studying patient or animal serum samples and screening neutralizing antibodies against CHIKV. Furthermore, the eGFP-CHIKV also has the potential to be used in large-scale and high-content assays.

## 2. Materials and Methods

### 2.1. Cell Lines, Viruses, Antibodies

**Figure 1:**
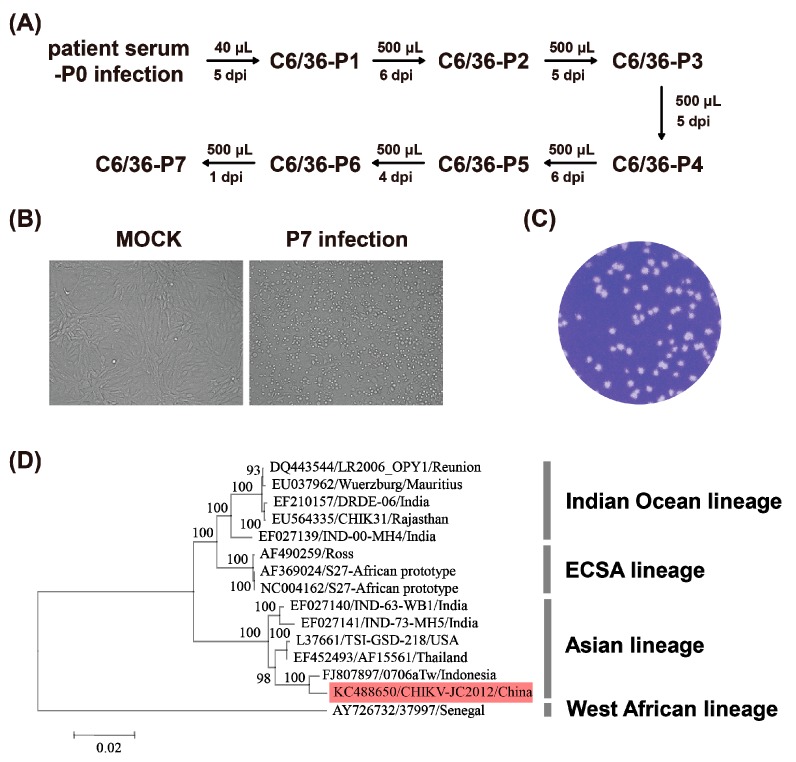
Isolation and characterization of CHIKV from clinical human cases. (**a**) the flow chart of virus isolation on C6/36 cells from human serum; (**b**) the CHIKV strain that was passaged for seven rounds on C6/36 cells showed apparent CPE on BHK-21 cells; (**c**) plaque morphology of the CHIKV strain on BHK-21 cells on the four days post-inoculation; and (**d**) phylogenetic analyses of CHIKV genome sequences using the neighbor-joining method. The newly isolated CHIKV strain is highlighted in red. ECSA lineage = the East, Central and South African lineage.

BHK-21 cells were cultured in Dulbecco’s modified Eagle’s medium (DMEM; Invitrogen, Darmstadt, Germany) with 10% Fetal Bovine Serum (FBS), 100 U/mL of penicillin and 100 μg/mL of streptomycin at 37 °C with 5% CO_2_. The mosquito cells of C6/36 *Aedes albopictus* clone were cultured in RPMI-1640 medium with 10% FBS at 28 °C. The CHIKV strain (GenBank accession No. KC488650) was isolated from a clinically CHIKV-positive patient in China through seven rounds of serial passages in C6/36 cells (Figure 1A). The CHIKV stock was stored as aliquots at −80 °C. This stock was used as parental CHIKV in all assays. The rabbit and mouse polyclonal antibodies against CHIKV E2 protein were generated by immunization of Japanese big-ear rabbits and BALB/C mice with SDS-PAGE purified CHIKV E2 protein, respectively.

### 2.2. Plasmid Construction

**Figure 2:**
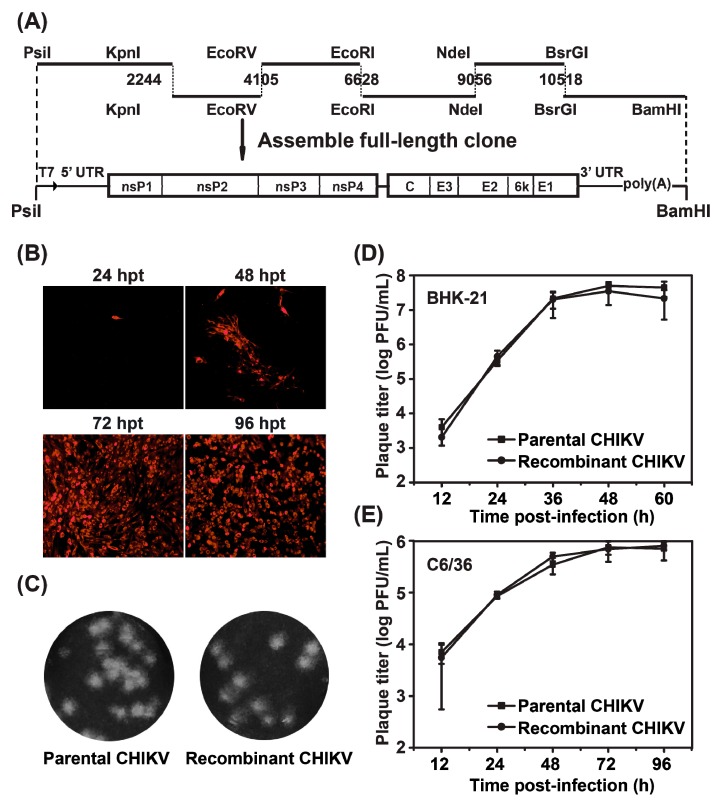
Construction and characterization of the full-length cDNA clone of CHIKV. (**a**) six cDNA fragments represented by thick lines were synthesized from genomic RNA through RT–PCR to cover the complete CHIKV genome and unique restriction sites as well as their nucleotide numbers are shown. Genome organization is depicted below. Individual fragments were assembled to form the full-length cDNA clone of CHIKV (pACYC-CHIKV). The complete CHIKV cDNA is under the control of T7 promoter elements for in vitro transcription. The numbers are the nucleotide positions based on the CHIKV genome sequences we identified in this study (KC488650); (**b**) IFA of viral protein expression in BHK-21 cells transfected with the full-length CHIKV RNA transcript. The transfected cells were analyzed by IFA at the indicated time points post-transfection; (**c**) plaque morphology of parental and recombinant CHIKV on BHK-21 cells; (**d**,**e**) comparison of the growth kinetics of recombinant and parental CHIKV in BHK-21 and C6/36 cells, respectively. The virus growth curves were compared at MOI = 0.1 on both cells. Three independent experiments were performed in duplicate, and the representative data were presented.

The viral RNA was extracted from the parental virus with QIAamp Viral RNA Mini Kit (Qiagen, Hilden, Germany) and used for reverse transcription PCR (RT-PCR). By using the SuperScript III One-Step RT-PCR System (Invitrogen), six cDNA fragments covering the complete viral genome of CHIKV were amplified from extracted viral RNA with six pairs of primers listed in Table 1, and then cloned into pACYC177 at PsiI and BamHI sites, yielding six subclones called CHIKV-A, CHIKV-B, CHIKV-C, CHIKV-D, CHIKV-E and CHIKV-F. Construct of CHIKV-A contained the sequence ranging from the 5′-end of the genome to the nucleotide position 2490 with a T7 promoter located before the 5′-end that was designed in primer CHIKV-5′UTR′-PsiI-F (Table 1). Constructs of CHIKV-B, CHIKV-C, CHIKV-D and CHIKV-E contained the sequences from nucleotide position 1877 to 4087, 4103 to 6802, 6604 to 9308 and 8713 to 10727, respectively. Construct of CHIKV-F included the sequence from nucleotide position 10,196 to the 3′-end with a poly(A) tail. The six subclones were assembled step-by-step into a full-length cDNA clone of pACYC-CHIKV following the scheme of the cloning strategy in Figure 2A.

**Figure 3:**
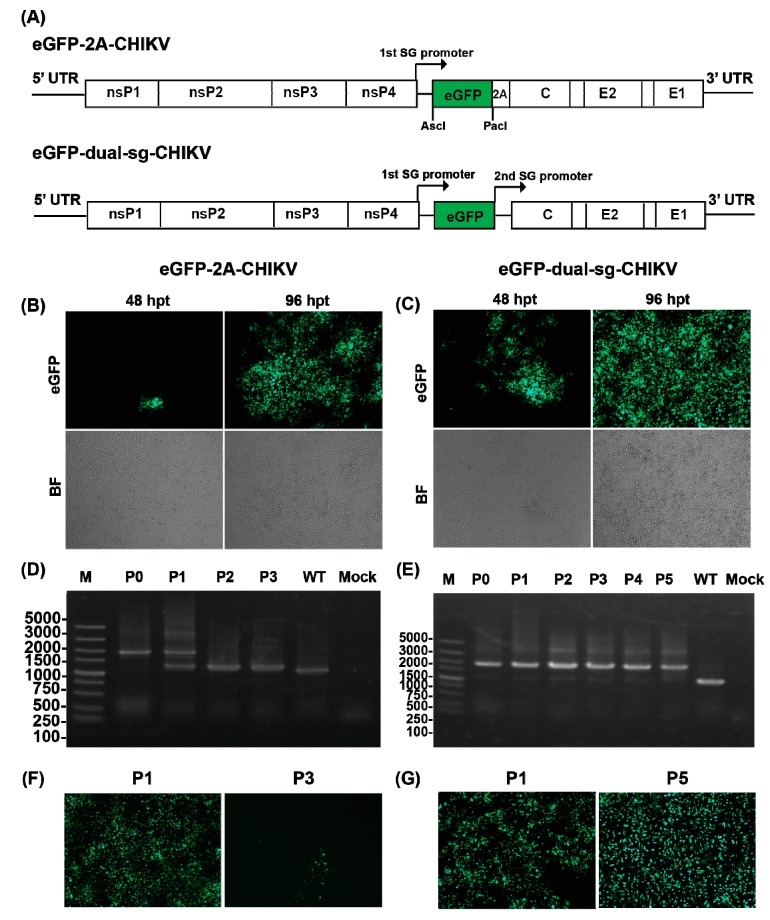
Construction and stability of CHIKV reporter viruses. (**a**) schematic of the construction of two versions of CHIKV reporter virus (eGFP-2A-CHIKV and eGFP-dual-sg-CHIKV). An infectious cDNA clone of pACYC-CHIKV was used as a backbone for the construction of CHIKV reporter viruses; (**b**,**c**) eGFP expressions in cells transfected with different versions of CHIKV reporter RNA transcripts. The data for eGFP-2A-CHIKV and eGFP-dual-sg-CHIKV were present on left and right panels, respectively, and thereafter. The expression of eGFP in transfected BHK-21 cells was analyzed by fluorescent microscopy at the indicated time points post-transfection. The lower panels were the images visualized under differential interference contrast microscopy; (**d**,**e**) detection of the eGFP gene during virus passage. Viral RNAs were extracted from culture supernatants of the indicated passages, respectively. RT–PCR was performed with primer pairs to cover the complete eGFP gene region. The resulting RT–PCR products were resolved by 1% agarose gel electrophoresis; and (**f**,**g**) detection of eGFP expression in BHK-21 cells infected with different passages of reporter viruses. eGFP expressions were observed under fluorescent microscope at 48 hpi. Both RT-PCR and eGFP expression results indicated that the reporter gene still maintained in eGFP-dual-sg-CHIKV. The eGFP-dual-sg-CHIKV was designated as eGFP-CHIKV.

The eGFP-CHIKV reporter virus cDNAs were constructed in multiple steps according to two different strategies as depicted in Figure 3A. Details for construction were as followed. In the first strategy, a cassette containing the AscI/PacI cleavage sites and FMDV 2A in reading frame of the structural polyprotein were introduced to pACYC-CHIKV by overlap PCR. Then, the eGFP gene was amplified and inserted into AscI and PacI sites to generate CHIKV reporter virus. In the second strategy, an expression cassette expressing a repeated sub-genomic (sg) promoter and eGFP were inserted between the nonstructural nsP4 gene and the 5′-terminal of structural genes. All primers used for PCRs were listed in Table 1. All constructs were confirmed by sequencing.

### 2.3. RNA Transcription and Transfection

Genome-length and reporting viral RNAs were prepared from the BamHI-linearized cDNA plasmids through in vitro transcription using mMESSENGER mMACHINE T7 Kit (Ambion, Austin, TX, USA). The RNAs were transfected into BHK-21 cells with DMRIE-C (Invitrogen). After transfection, supernatants were collected at different time points. The culture medium containing viruses were aliquoted and stored at −80 °C for the next experiments.

### 2.4. Plaque Assay

BHK-21 cells were seeded into 24-well plates at a density of 1 × 10^5^ cells per well one day before plaque assay. A series of 1:10 dilutions were made by mixing 15 μL of virus sample with 135 μL of DMEM. Then, 100 μL of each dilution were added to individual wells of 24-well plates containing confluent BHK-21 cells. The plates were incubated at 37 °C with 5% CO_2_ for 1 h before the layer of 2% methyl cellulose was added. After 3 days of incubation at 37 °C with 5% CO_2_, the cells were fixed with 3.7% formaldehyde and then stained with 1% crystal violet. Plaque morphology and numbers were recorded after washing the plates with tap water.

### 2.5. Immunofluorescence Assay (IFA)

BHK-21 cells transfected with the CHIKV genome-length RNA were seeded on Chamber Slide (Nalge Nunc, Rochester, NY, USA). At 24, 48, 72 and 96 h post-transfection (hpt), the transfected cells were collected, washed with PBS and fixed by cold (−20 °C) 5% acetic acid in acetone for 15 min at room temperature. The fixed cells were washed with PBS and incubated with CHIKV E2 polyclonal serum from rabbit (1:250 dilution with PBS) for 1 h. After washing with PBS, the cells were then incubated with goat anti-rabbit IgG conjugated with Texas-Red (Proteintech, Wuhan, China) at a 1:125 dilution with PBS at room temperature for another hour. The cells on the slide were mounted with 90% glycerol and examined under a fluorescent microscope. The fluorescent signal images were taken at 200× magnification with a NIKON upright fluorescence microscope (Tokyo, Japan).

### 2.6. Antiviral Assay of eGFP-CHIKV by Ribavirin

One day prior to infection, BHK-21 cells were seeded in 12-well plates at a density of 1 × 10^5^ cells per well. Then, the cells were inoculated with 0.2 mL of diluted recombinant CHIKV and eGFP-CHIKV virus (at a multiplicity of infection of 0.05) and incubated at 37 °C with 5% CO_2_ for 1 h. Subsequently, the culture was replaced with fresh medium containing different concentrations of ribavirin. After incubation at 37 °C for 48 h, the supernatants were collected and viral titers were quantified by plaque assay. Antiviral activity of ribavirin was expressed as the 50% effective concentration (EC_50_) and calculated as described previously [23].

### 2.7. Neutralization Assay Based on eGFP-CHIKV Reporter Virus

The neutralizing activities of the serum samples from CHIKV-infected patients and mice were determined using eGFP-CHIKV reporting virus in BHK-21 cells in triplicates. Briefly, BHK-21 cells were seeded in 24-well plates at a density of 8 × 10^4^ cells per well. The following day, eGFP-CHIKV were used in the NT assay at an MOI of 0.05. eGFP-CHIKV were incubated with 4-fold serial dilutions of heat-inactivated patient sera (starting at 1:10 dilution) and mouse sera or CHIKV E2 polyclonal antibody (starting at 1:20 dilution) for 1 h at 37 °C before adding the mixture to the monolayer of BHK-21 cells in the 24-well plates. Simultaneously, the serum from health person was used as a negative control. After incubation for another 1 h at 37 °C, the inoculum was removed. Cells were washed with PBS and fresh medium was added to each well. We examined the level of neutralization activity by measurement of eGFP fluorescence intensity at 48 h post-infection (hpi) using both fluorescent microscopy and microplate fluorimeter. The percentage of infectivity was calculated as: %Infectivity = (fluorescence intensity from serum samples / fluorescence intensity from negative control samples). The experiments were independently repeated three times.

### 2.8. Plaque Reduction Neutralization Tests (PRNTs)

PRNT assay was carried out to detect and quantify the presence of neutralizing antibodies in the human serum samples as previously described [24]. In brief, about 100 PFU of infectious WT CHIKV were incubated with 100 μL of 4-fold serial dilutions of heat-inactivated serum samples (starting at 1:40 dilution) at 37 °C for 1 h. The virus-serum mixture was then inoculated to BHK-21 cell monolayer cultured in 12-well plates and incubated at 37 °C for 1 h with rocking every 15 min. Next, the supernatant was removed, and the layer of 2% methyl cellulose was added. After further incubation at 37 °C with 5% CO_2_ for 2 days, the cells were fixed and plaque numbers were recorded as described above (section 2.4). WT CHIKV alone without serum incubation was served as negative control. The percentage of infectivity was calculated as: %Infectivity = (number of plaques from serum samples / number of plaques from negative control).

### 2.9. Real-Time RT-PCR Analysis

To detect and quantify the genome copy numbers of WT CHIKV, SYBR Green based real time RT-PCR analysis was used. Briefly, 100 μL of WT CHIKV corresponding to MOI of 1 were mixed with 100 μL heat-inactivated serum samples (1:40 dilution). After being incubated at 37 °C for 1 h, the virus-serum mixture was added to BHK-21 cell monolayer in 12-well plates. After 1 h of incubation at 37 °C, unbound virus was removed by three washes with cold PBS. The total cellular RNAs were extracted using Trizol reagent (TaKaRa, Dalian, China). Real time RT-PCR was performed using one step SYBR green PrimeScript PLUS RT-PCR kit (TaKaRa). The primer set was designed to amplify a 81 bp-long region within the E2-6K-E1 gene as described previously [25]. The primer sequences were CHIKV-9923-F (forward) and CHIKV-10003-R (reverse) (Table 1). Amplification conditions were as follows: 42 °C for 5 min, 95 °C for 10 s and 40 cycles of 95 °C for 5 s, 60 °C for 34 s. A melting curve analysis was performed to verify the authenticity of the amplification after each reaction. In vitro transcribed RNA were used to establish standard curve and the genomic RNA copies were determined from the respective C_T_ values based on the standard curve.

### 2.10. Statistical Analysis

The Student’s *t*-test and ANOVA test were used to determine if there were significant differences (*p* < 0.05) for all tested viruses. The statistical analyses were performed in IBM SPSS Statistics v18.0 (Chicago, IL, USA).

## 3. Results

### 3.1. Isolation and Characterization of CHIKV

The CHIKV strain (KC488650) used for the construction of infectious clone was isolated from a clinical sample as depicted in Figure 1A. The isolated CHIKV replicated efficiently and caused typical cytopathic effects (CPE) on BHK-21 cells (Figure 1B) with a high viral titer of approximately 1.5 × 10^7^ PFU/mL determined by plaque assay (Figure 1C). The viral complete genome sequencing and the phylogenetic analysis indicated that this newly isolated strain belonged to the Asian lineage of CHIKV. Our isolate was sister to the strain 0706aTw isolated from Indonesia in 2007 (Figure 1D) and two isolates shared high identity of their nucleotide sequences up to 99.4%.

### 3.2. Construction and Characterization of of the Full-Length CHIKV cDNA Clone

The full-length CHIKV cDNA clone, pACYC-CHIKV, was assembled with six subclones through distinct restriction cut sites as indicated in Figure 2A. A T7 promoter was engineered at the 5′-end of viral genome sequence for in vitro transcription. The recombinant CHIKV genomic RNA was in vitro transcribed from CHIKV cDNA clone, followed by linearization with BamHI. In vitro transcribed viral RNA was transfected into BHK-21 cells and IFA was firstly used to demonstrate viral replication by detecting viral E2 protein expression (Figure 2B). Increasing number of IFA-positive cells expressing viral E2 protein was observed from 24 to 96 hpt and almost 100% cells were IFA-positive at 72 and 96 hpt. Then, plaque morphologies were compared between the recombinant and parental viruses. As shown in Figure 2C, the plaque size of recombinant virus is similar to that of parental virus. Consistently, indistinguishable viral growth kinetics at MOI of 0.1 were observed between recombinant and parental viruses in either BHK-21 or C6/36 cells (Figure 2D,E). Overall, our data demonstrated that CHIKV infectious clone was constructed successfully and recombinant virus was infectious in both mosquito and mammalian cells. The recombinant CHIKV derived from infectious clone is designated as WT CHIKV in this paper.

### 3.3. Construction of the CHIKV Reporter Virus with eGFP

Two strategies were used to construct eGFP-CHIKV reporter viruses as shown in Figure 3A. The first strategy is that eGFP gene was inserted before the structure protein of capsid (C). In order to release the eGFP from capsid, FMDV 2A for autocleavage was introduced between them. The construct was designated as eGFP-2A-CHIKV. A dual subgenomic (sg) promoter method was used based on the second strategy to construct reporter virus that was called eGFP-dual-sg-CHIKV. Additional sg promoter was used for eGFP cassette expression that was inserted before structure genes.

Equal amounts of in vitro transcribed genome-length RNAs of eGFP-2A-CHIKV and eGFP-dual-sg-CHIKV were transfected into BHK-21 cells to compare their viral replication capacity. The expression levels of eGFP were monitored under a fluorescent microscope for these two constructs of reporter viruses. As demonstrated in Figure 3B,C, eGFP-dual-sg-CHIKV produced more positive eGFP cells than eGFP-2A-CHIKV at each time point post-transfection. Furthermore, more CPE were also observed in transfected cells for eGFP-dual-sg-CHIKV (Figure 3C), which indicated a replication advantage of eGFP-dual-sg-CHIKV over eGFP-2A-CHIKV (Figure 3B).

### 3.4. Characterization of the CHIKV Reporter Virus

After demonstrating that both reporter viruses are replication-competent, their stabilities were tested in cell culture. The viruses in supernatants from reporter virus RNA transfected BHK-21 cells were defined as P0 passage and were used for blinding passage on BHI-21 cells. The viruses from each passage were defined as P1 to P5 passages. Viral RNAs were first extracted from P0 to P3 passages of eGFP-2A-CHIKV and P0 to P5 passages of eGFP-dual-sg-CHIKV, respectively. RT-PCRs were performed to amplify the fragment between nsP4 and capsid that covers the region of the inserted reporter gene using primers CHIKV-7376-F and CHIKV-8498-R (Table 1). Different sizes of bands should be detected from RT-PCR products of WT (~1.5 Kb) and reporter viruses (~2 Kb) (Figure 3D,E). For eGFP-2A-CHIKV, reporter genes began to lose from P1 passage as indicated by two RT-PCR products of both 1.5 Kb and 2 Kb fragments, and were completely lost at the P2/P3 passage (Figure 3D). For eGFP-dual-sg-CHIKV, only specific 2 kb RT-PCR products were observed from P0 to P5 passages, which indicated eGFP-dual-sg-CHIKV reporter virus is much more stable than eGFP-2A-CHIKV (Figure 3E). Consistent with RT-PCR results, a high eGFP expression level was detected in BHK-21 cells infected with P5 of eGFP-dual-sg-CHIKV, but only sporadic eGFP positive cells were observed in P3 of eGFP-2A-CHIKV infected cells (Figure 3F,G). Our results demonstrated that eGFP-dual-sg-CHIKV reporter virus was stable and designated as eGFP-CHIKV in the following study.

**Figure 4:**
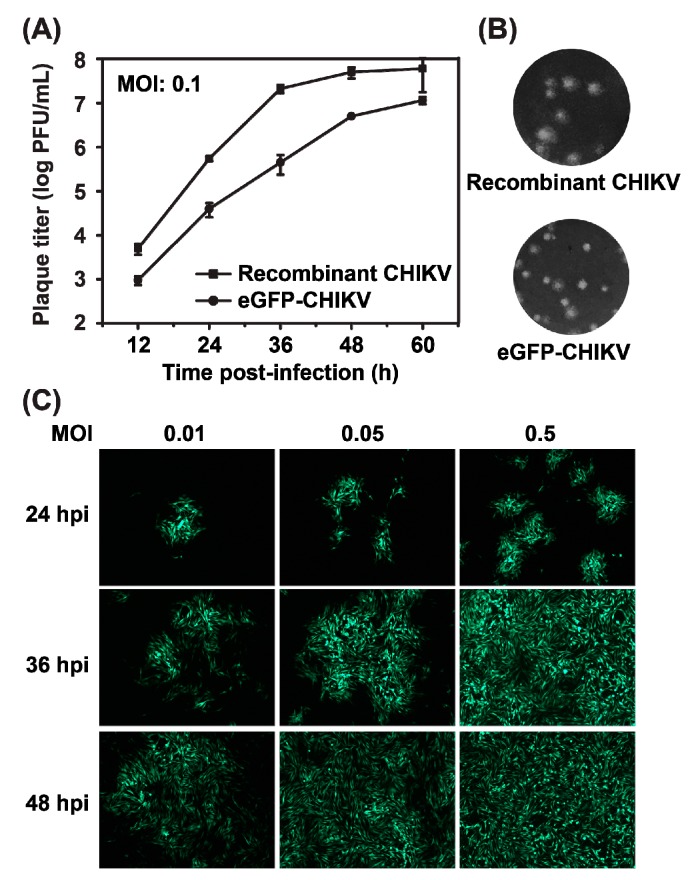
Characterization of eGFP-CHIKV reporter viruses. (**a**) comparison of the growth kinetics of recombinant WT CHIKV and eGFP-CHIKV in BHK-21 cells. The representative data were presented from three independent experiments; (**b**) plaque morphology of recombinant WT CHIKV and eGFP-CHIKV on BHK-21 cells; and (**c**) correlation between eGFP expression and different MOIs infection. BHK-21 cells were infected with eGFP-CHIKV at the indicated MOI and eGFP expression were observed at 24, 36 and 48 hpi.

Plaque morphology and viral growth curve were compared between WT CHIKV and eGFP-CHIKV reporter virus. As measured by plaque assay, eGFP-CHIKV could replicate efficiently although its viral titer was about 10-fold lower than that of WT CHIKV (Figure 4A). eGFP-CHIKV displayed a smaller plaque size than WT CHIKV (Figure 4B). To determine whether the level of eGFP expression is correlated with the efficiency of viral replication, different MOIs of eGFP-CHIKV were used to infect naïve BHK-21 cells, and the numbers of eGFP-positive cells were recorded at different time points post-infection. A time- and dose-dependent increase in the number of eGFP-positive cells was observed (Figure 4C), which indicated that eGFP expression levels could be used to monitor and quantify viral replication. We used MOI of 0.05 for the following experiments as almost 100% eGFP-positive cells were observed at 48 hpi. Overall, the results show that the eGFP-CHIKV reporter virus is replication-competent, stable and infectious.

### 3.5. Inhibitory Effects of Ribavirin on eGFP-CHIKV Reporter Virus

**Figure 5:**
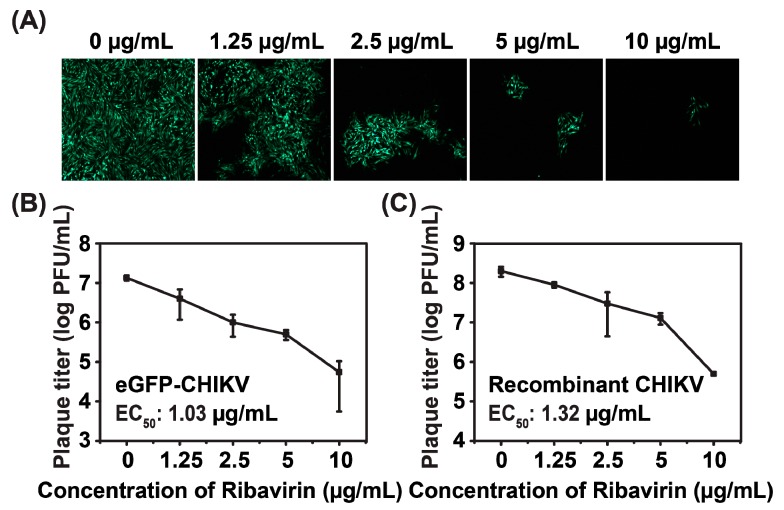
Antiviral activity of ribavirin on eGFP-CHIKV reporter viruses. (**a**) detection of eGFP expression by fluorescent microscope at different concentrations of ribavirin; (**b**) viral titer reduction assay of CHIKV with different concentrations of ribavirin. BHK-21 cells were infected with eGFP-CHIKV at MOI of 0.05 and treated with various concentrations of ribavirin. The viral titers were quantified by plaque assay at 48 hpi; and (**c**) viral titer reduction assay of WT CHIKV at different concentrations of ribavirin. The experiments were performed in triplicate, and the representative data were presented.

It has been demonstrated that ribavirin could inhibit CHIKV replication [26,27]. To further confirm the correlation between eGFP expression and viral replication, we assessed the antiviral activity of ribavirin using eGFP-CHIKV reporter virus. The eGFP expression levels and virus titers were determined at different concentrations of ribavirin that did not cause significant cytotoxic effects. The maximum concentration of ribavirin was 10 μg/mL and 2-fold serial dilutions were used in this study. The eGFP expressions of infected cells were reduced in a dose-dependent manner (Figure 5A), which was consistent with the reduction of viral titers (Figure 5B). Furthermore, the EC_50_ of ribavirin for eGFP-CHIKV was 1.03 μg/mL (4.22 μM), which was similar to that of WT CHIKV, i.e., EC_50_ = 1.32 μg/mL (5.41 μM) (Figure 5C). The results further confirmed that eGFP expression could be used to surrogate the replication of WT CHIKV. In addition, it was also suggested that the eGFP-CHIKV reporter virus system could be used for screening potential anti-CHIKV inhibitors.

### 3.6. Confirmation of Neutralization Assay Using eGFP-CHIKV with CHIKV Patients’ Sera

**Figure 6:**
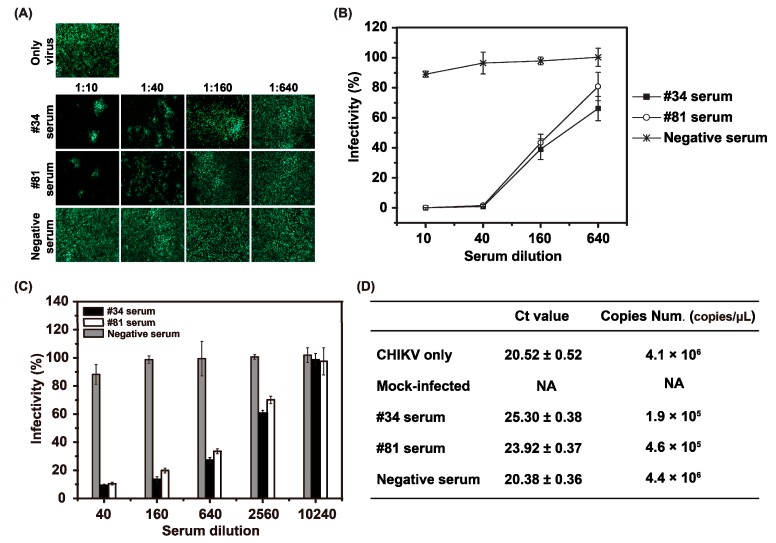
Confirmation of the availability of eGFP-CHIKV in neutralization assay with the serum samples from CHIKV-infected patients. (**a**) detection of eGFP expression by fluorescent microscope with different dilutions of human sera. The neutralization assay was performed in a 12-well plate. Serially 4-fold diluted human sera were incubated with eGFP-CHIKV at an MOI of 0.05 for 1 h at 37 °C before adding the mixture to the monolayer of BHK-21 cells in the 24-well plates. The readout of eGFP expression was recorded at 48 hpi. The serum from health person was used as a negative control; (**b**) quantification of neutralization assay with increasing serum dilution. The percentage inhibition of infectivity was normalized to eGFP-CHIKV infection without serum incubation by quantification of eGFP expression levels; (**c**) neutralizing activity of the human sera against WT CHIKV based on PRNT assay. WT CHIKV was incubated with 4-fold dilutions of individual sera before infecting BHK-21 cells. The percentage inhibition of infectivity for each dilution was normalized to WT CHIKV infection alone by counting the number of plaque forming units (PFU); (**d**) detection of CHIKV genome copy number in BHK-21 cells treated with virus or virus-antiserum mixture by real-time RT-PCR. The results are positive if Ct value ≤34, otherwise negative if Ct value >34. The genome copy numbers of #34 and #81 were significantly decreased than those of control samples (*t*-test, *p* < 0.05). CHIKV only = BHK-21 cells were infected by WT CHIKV without serum incubation; mock-infected = BHK-21 cells were neither infected by WT CHIKV nor incubated with serum; NA = data not available. All experiments were performed in triplicate, and the representative data were presented.

Neutralization assay with eGFP-CHIKV were firstly evaluated by using human sera from two CHIKV-infected patients (#34 and #81), which had been confirmed to be positive against CHIKV. The eGFP-CHIKV was mixed with the respective serum dilutions and incubated in BHK-21 cells. At 48 hpi, the numbers of eGFP-positive cells were recorded to determine the infectivity rates. As shown in Figure 6A, neutralizing activities were observed after incubations with CHIKV sera (#34 and #81) at different dilutions. There was little or no effect observed when eGFP-CHIKV was incubated with the negative control of a healthy human serum (Figure 6A), demonstrating that neutralization is specifically induced by the sera against CHIKV. Furthermore, the two CHIKV-positive human sera samples significantly reduced the percentage of infectivity of eGFP-CHIKV reporter viruses in a dose-dependent manner with quantification of eGFP positive cells (Figure 6B). In order to assess the efficacy of eGFP-CHIKV on neutralizing assay, the conventional PRNT assay was conducted. Similarly, there was an excellent correlation between the plaque reduction and the dilutions of neutralizing human sera (Figure 6C). Both dilution 1:40 of #34 and #81 sera could inhibit approximately 90% of infectious CHIKV in PRNT assay (Figure 6C), which was consistent with that of neutralization assay based on eGFP-CHIKV (Figure 6B). The percentages of infectivity of WT CHIKV was lower than 50% when sera #34 and #81 were diluted to 1:160 and 1:640 (Figure 6C). No significant differences in inhibition were observed between dilution 1:10240 of these two human sera and negative human serum (ANOVA test, *p* > 0.05). As control, the changes in CHIKV genome copies following the co-incubation of human sera and WT CHIKV in fusion/entry step were monitored by real-time RT-PCR analysis. The CHIKV genome copies can be decreased by nearly 22-fold and 9-fold when 1:40 dilutions of #34 and #81 sera were used, respectively (Figure 6D). We thus concluded that the ability of serum neutralization could be measured by eGFP expression with eGFP-CHIKV reporter viruses that are visible and suitable for the neutralization test.

### 3.7. Neutralization Assay with Sera from Mice Using eGFP-CHIKV

**Figure 7:**
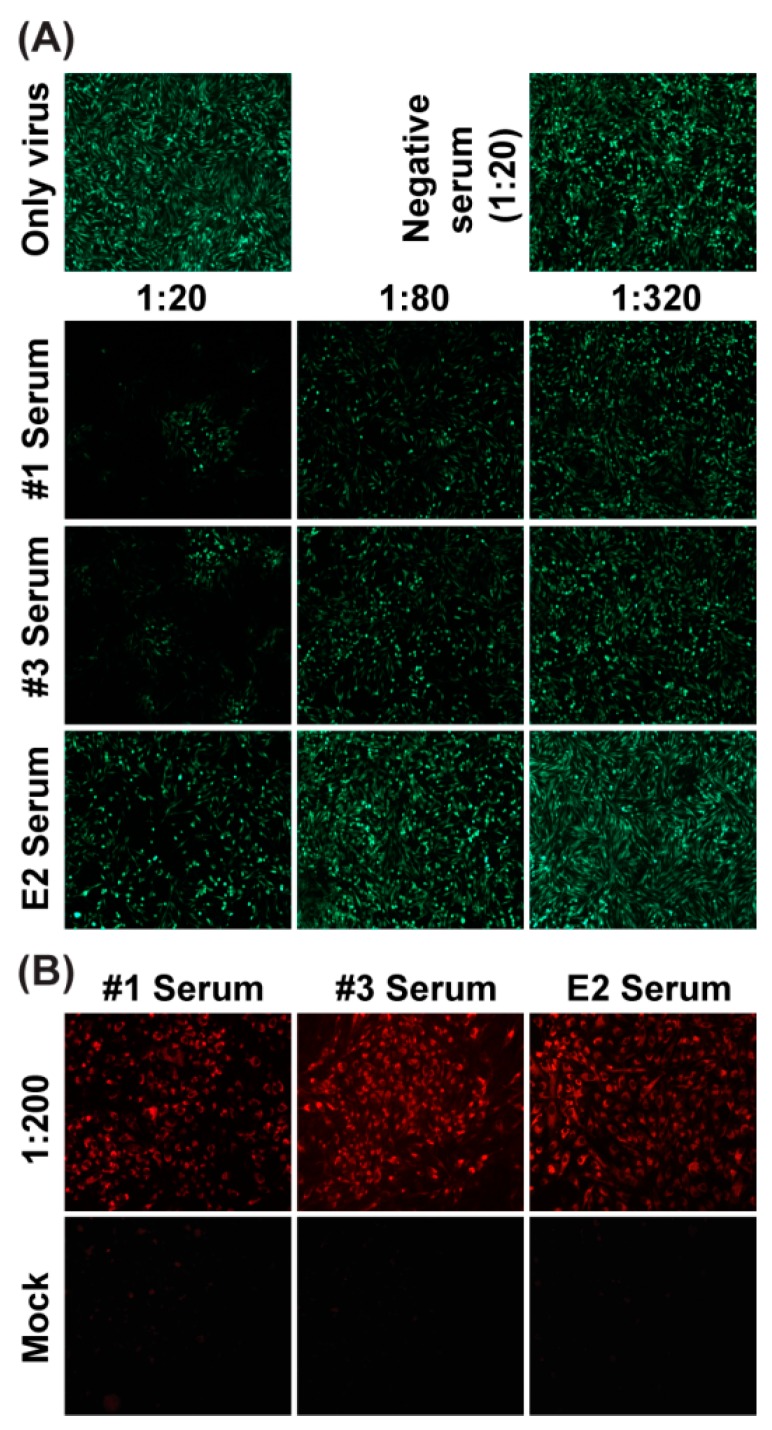
Neutralization assay with sera from mice using eGFP-CHIKV. (**a**) detection of eGFP expression by fluorescent microscope with different dilutions of sera from mice immunized with different antigens. #1 and #3 sera were from two mice immunized with formalin-inactivated CHIKV, and anti-E2 serum was obtained from one mouse immunized with SDS-PAGE purified CHIKV E2 protein. NT assay was performed as described for CHIKV-infected patient sera; (**b**) the reactivity of the tested sera against CHIKV through an IFA. All three sera (#1, #3 and E2 sera) were diluted at 1:200. Mock and CHIKV infected BHK-21 cells were used to performed IFA.

The serum samples collected from two mice immunized with formalin-inactivated CHIKV (#1 and #3 sera) and one mouse immunized with SDS-PAGE purified CHIKV E2 protein (anti-E2 serum) were analyzed with the NT assay. The eGFP-CHIKV was mixed with 4-fold serially diluted serum and incubated with cells for 48 h. All pre-immunization sera were negative for anti-CHIKV neutralizing antibodies and one of representative data at 1:20 dilution was shown in Figure 7A as a negative control. A dose dependence of eGFP positive cells were observed for both #1 and #3 sera from dilution 1:20 to 1:320 (Figure 7A). In contrast, there was only a minor effect on eGFP expression for anti-E2 serum at 1:20 dilution. To further confirm the reactivity of the tested sera against CHIKV, an IFA (Figure 7B) was performed using CHIKV-infected cells. All three sera (#1, #3 and anti-E2 sera) were found to be reactive against CHIKV as IFA positive cells were observed in CHIKV infected cells. At the same time, no positive cells were found in mock infected cells for all tested sera, which excluded the possibility of false positive results. Additionally, the antibody titers of #1, #3 and anti-E2 sera were assessed by ELISA. The coating antigen for ELISA was purified recombinant E2 protein. The results revealed that anti-E2 sera yielded the maximum antibody titers. Overall, the results demonstrated that our NT assay based on eGFP-CHIKV could easily differentiate neutralizing serum/antibody against CHIKV from non-neutralizing serum/antibody with E2 protein.

## 4. Discussion

CHIKV, a member of the *Alphavirus* genus, has re-emerged as a major threat to global public health and there is an urgent need for continued research into the epidemiology, pathogenesis, prevention, and treatment of CHIKV infections [2]. In recent years, significant progress has been made in our understanding of the replication mechanism of alphavirus [28,29]. These mechanism studies further prompted development of reverse genetic for alphavirus [4,30,31,32,33,34,35]. For CHIKV, different infectious clones and reporter viruses have been also developed to follow up viral replication both in vitro and in vivo [27,31,36,37,38]. In general, the CHIKV strain La Réunion (Indian Ocean lineage, Figure 1D) isolated from infected humans is commonly used for reporter virus system and functional research [37,38,39]. In our study, the new infectious clone was derived from a Chinese human isolate (strain CHIKV-JC2012) that belongs to the Asian lineage (Figure 1D). As we know, lineage-specific adaptive mutations could accumulate in CHIKVs from different genetic lineages, which may affect the infectivity range and epidemic dynamics [7,40]. Thus, recombinant and reporter CHIKVs derived from different lineages could expand the range and diversity of CHIKV related analyses and applications.

In this study, we developed an NT assay using the eGFP-CHIKV reporter virus to evaluate neutralizing activity of sera/antibody against CHIKV. On one hand, we constructed an infectious clone of CHIKV and a eGFP-CHIKV reporter virus based on the isolate in our lab (strain CHIKV-JC2012) (Figure 1). The recombinant CHIKV and reporter CHIKV are replication-competent and infectious (Figure 2). The expression level of eGFP correlated with viral replication, which enabled us to perform neutralization assay using eGFP-CHIKV to quantify neutralizing antibody/serum against CHIKV. We used this reporter virus to detect the neutralizing activities of the serum samples from two CHIKV-infected patients, and demonstrated that the replication of eGFP-CHIKV was inhibited in a dose-dependent manner. On the other hand, the conventional PRNT assay and real-time RT-PCR analysis were conducted to further confirm the suitability and reliability of eGFP-CHIKV based NT assay. Although the PRNT exhibited more efficient ability to inhibit the infectious CHIKV, we speculated that the initial amounts of CHIKV particles could account for the difference of performance between PRNT and eGFP-CHIKV based NT assay (MOI, 0.0005 vs. 0.05) as less viruses are likely to be more efficiently reduced by neutralizing antibody. Overall, the newly developed NT assay based on eGFP-CHIKV reporter viruses is suitable for neutralization test.

In our assay, we confirmed in a previous study that inactivated CHIKV represents a good immunogen for inducing neutralizing antibodies (Figure 7A) comparing with denaturing recombinant E2 protein although mice immunized with denaturing E2 protein produced highest antibody titer by ELISA. However, we cannot exclude the possibility that these antibodies from denaturing E2 protein immunized-mice are against the same denatured epitope with the coating antigen of ELISA, while inactivated CHIKV-immunized sera are likely against other conformational epitopes. Nonetheless, our results demonstrated that, although ELISAs are very fast and easy to perform for measuring and deciding the absence or presence of antibodies, ELISAs could not differentiate neutralizing and non-neutralizing antibodies. In summary, NT assay is the best way to evaluate virus-neutralizing antibodies biologically that will produce accurate and specific results.

When eGFP-CHIKV reporter viruses are used for NT assays, there are some advantages compared with VRP and pseudotyped lentiviral vectors [21,22]. Firstly, the production of eGFP-CHIKV reporter viruses is technically easy as the reporter viruses are quite stable and infectious. The growth curve of eGFP-CHIKV is comparable with that of WT CHIKV. Large amounts of eGFP-CHIKV could be obtained from infected cells once the reporter viruses were recovered from cells transfected with transcribed recombinant RNA. In contrast, a new cycle of transfection is required each time to get new batches of CHIKV VRPs or CHIKV-lentiviral-vectors, which are tedious and technically difficult. Secondly, the assays are not expensive, as no additional reagents are needed. The readouts of the NT assay based on eGFP-CHIKV reporter viruses were performed with a fluorescence microscope by direct observation of eGFP expression, which is much easier than conventional methods. Lastly, eGFP-CHIKV also has the high throughput potential by using high content assays with quantification of eGFP positive cells. It will omit any further plate processing procedures, such as substrate addition to produce different signals for readout, cell fixation, plate washing or cell lysis, which will further decrease the cost and complexity of the screening process. Moreover, the eGFP-CHIKV reporter virus derived from an Asian lineage could provide opportunities for the comparison study of antiviral activity of neutralizing antibodies and compounds among different lineages in a high-throughput manner. In this regard, the relationships between virulence, resistance, and viral fitness of CHIKV strains from distinct phylogenetic groups could be further investigated.

Comparing NT assays using the CHIKV VRP and pseudotyped lentiviral vectors, one disadvantage for eGFP-CHIKV is the biosafety issue as CHIKV is classified as a biosafety level 3 (BSL-3) pathogen. This problem may be resolved by using attenuated vaccine candidates to create eGFP-CHIKV reporter viruses. Recently, various live-attenuated CHIKV vaccine candidates have been developed through inserting an IRES (internal ribosome entry site) sequence into the genome of CHIKV [14] or deleting a large part of the gene encoding nsP3 or the entire gene encoding 6K [13]. These live vaccine candidates may help develop new versions of CHIKV reporter viruses to increase safety in the future.

## 5. Conclusions

In conclusion, the eGFP-CHIKV reporter virus constructed in our study is stable and efficient for the detection and quantification of CHIKV replication. The neutralization assay based on this reporter virus is demonstrated to be rapid and specific for evaluating neutralizing antibodies of viruses. Importantly, the eGFP-CHIKV reporter virus derived from a new Asian strain, which was rarely reported previously, could facilitate the systematic studies among different lineages of CHIKV in a high-throughput manner.

## Figures and Tables

**Table 1:** Primer sequences.

Primer	Sequence
CHIKV-5′UTR′-PsiI-F	cgcTTATAATAATACGACTCACTATAGatggctgcgtgagacacacg
CHIKV-nsP2-BamHI-R(2490)	cgcGGATCCctcaccaaggcgatcaaggc
CHIKV-nsP2-PsiI-F(1877)	cgcTTATAACGTACGATGGCCGAGTCCTAGTG
CHIKV-nsP3-BamHI-R(4087)	cgcGGATCCgatatccatgcgttttacccggtac
CHIKV-nsP3-PsiI-F(4103)	cgcTTATAAgatatcGCGAAGAACGATGAAGAG
CHIKV-nsP4-BamHI-R(6802)	cgcGGATCCgcattaaggcggtaagcgca
CHIKV-nsP4-PsiI-F(6604)	cgcTTATAACTTGGCAACAGCGTACCTATG
CHIKV-E2-BamHI-R(9308)	cgcGGATCCtgcatgtcacatttgccagag
CHIKV-nsP2-PsiI-F(8713)	cgcTTATAACATGATTGGACCAAGCTGCG
CHIKV-E1-BamHI-R(10727)	cgcGGATCCggtgctgtgtgctgcagcgacg
CHIKV-E1-PsiI-F(10196)	cgcTTATAAGCCTACCTGATTACAGC
CHIKV-3′UTR-BamHI-R	cgcGGATCCACTAGTTTTTTTTTTTTTTTTTTTTTTTTTTTTTTTTTTTTTgaaatatt
CHIKV-AscI-PacI-2A-F	TAAATACCAATCAGCCATAcggcgcgccaagACATACgccttaattaatCAGCTGTTGAATTTTGACCTTCTCAA
CHIKV-AscI-PacI-2A-R	GGTTGGGATAAACTCCATTGGCCCAGGGTTGGACTCGACGTCTCCCGCCAGCTTGAGAAGGTCAAAATTCAACA
CHIKV-PacI-2SG promoter-F	gccttaattaatGTCATAACCTTGTACGG
CHIKV-2SG promoter-C-F	AATCAGCCATAATGGAGTTTATCCCAACC
CHIKV-2SG promoter-C-R	ATAAACTCCATTATGGCTGATTGGTATTT
eGFP-AscI-F	ttggcgcgccatggtgagcaagggcgaggag
PacI-eGFP-stop-R	TccttaattaaCTActtgtacagctcgtccatgcc
CHIKV-7376-F	gaagtgcagggtatatcag
CHIKV-8498-R	gatgcttgtagcagctgat
CHIKV-9923-F	TGAGCGTCGGTGCCCAC
CHIKV-10003-R	GAGTCTTATACCGTACTCCCACCGT

## Abbreviations

CHIKV	Chikungunya virus
mAbs	monoclonal antibodies
NT	neutralization
MOI	multiplicity of infection
eGFP	enhanced green fluorescent protein
Gluc	Gaussia luciferase
